# Effects of private caregivers on nutritional risk and anxiety in stroke survivors with dysphagia: an observational study

**DOI:** 10.3389/fnut.2025.1513609

**Published:** 2025-05-30

**Authors:** Weijia Zhao, Jing Zeng, Shuaiqi Li, Sheju Chen

**Affiliations:** ^1^School of Public Health, Zhengzhou University, Zhengzhou, China; ^2^Department of Rehabilitation Medicine, The First Affiliated Hospital of Zhengzhou University, Zhengzhou, China; ^3^Health Department, The Third Affiliated Hospital of Zhengzhou University, Zhengzhou, China

**Keywords:** accompanying persons, accompany nursing staff, professional escorts, patient escorts, rehabilitation

## Abstract

**Background:**

Private caregivers are common in developing countries as supplements to healthcare human resources. However, the effects of private caregivers on inpatient conditions remain unclear. This study explored the effect of private caregivers on nutritional risk and anxiety in patients with dysphagia after ischemic stroke.

**Methods:**

This observational study included patients with dysphagia after ischemic stroke between 2022 and 2024 in central China. Participants were divided into non-caregiver and private caregiver groups. A single-factor analysis was used to explore the differences between the baseline assessments of the two groups. We then used propensity score matching (PSM) to balance significant baseline variables, including anxiety at admission, and demographic and clinical characteristics. In the matched sample, we used the average treatment effect on the treated (ATT) to uncover the influence of the patient groups on nutritional risks and anxiety on day 10.

**Results:**

A total of 2,234 patients were included, and there were 766 cases in the private caregiver group. Before PSM, there were significant reductions in anxiety (47.00 vs. 32.86%, *P* < 0.001) and nutritional risks (100.00 vs. 70.55%, *P* < 0.001) from days 1 to 10. After PSM, there were no significant differences in any baseline assessments, and 766 pairs of cases were successfully captured. The ATT analysis showed that before and after PSM, there was no significant correlation between groups and nutritional risks (30.75 vs. 29.41%, *P* = 0.573), but there was a significant correlation between groups and anxiety on day 10 (34.89 vs. 23.40%, ATT = 0.234 after matching, *P* < 0.001).

**Conclusions:**

Private caregivers can effectively reduce anxiety in hospitalized patients with dysphagia following ischemic stroke, but they do not have a significant impact on nutritional risks.

## 1 Introduction

Ischemic stroke typically causes functional impairment to various degrees and types, with dysphagia being one of them (1). Swallowing disorders can lead to malnutrition, pneumonia, choking, and decreased quality of life (2). According to previous studies, almost half of the patients experience swallowing issues during the acute and subacute phases of stroke (3). Additionally, stroke increases the risk of psychological problems (4). It has been reported that ~30% of stroke survivors suffer from generalized anxiety (5). Therefore, the care of stroke patients requires dual attention to both physiological and psychological aspects (6).

In some developing countries, such as China and India, the lack of nursing staff has forced patients' families to take on some caregiving responsibilities (7). However, most family members do not receive relevant training and lack nursing experience, which can result in suboptimal outcomes. In this context, a role has emerged between professional caregivers and family members known as private caregivers or professional escorts (8). These practitioners can be regarded as supplements and compromises to healthcare human resources (9). The terminology and scope of work for private caregivers can vary across regions. In China, they are referred to as accompanying caregivers. Most of these service providers do not have a medical background, but are trained to offer assistance in daily living, exercises, procedures, toileting, and personal hygiene for patients during hospitalization (10). In China, the costs of private caregiving are primarily paid by patients and are excluded from most medical insurance coverage. Therefore, private caregiving may increase the financial burden on patients to some extent, while alleviating the pressure on public health insurance (11). On this basis, self-funded private care might indicate higher socioeconomic status.

Due to the professional experience and training, private caregivers may be able to improve treatment outcomes (12). Some studies have reported that caregivers can positively influence the success rate of tracheostomy tube removal in patients with persistent vegetative state (13). For conscious patients, private caregivers can provide companionship and communication, offering comfort and encouragement during treatment (10). This may help to improve patients' psychological conditions.

Malnutrition is a risk factor for rehabilitation outcomes in stroke survivors with dysphagia (14). To address this issue, enteral nutritional support is clinically practiced (15). This nutritional method deprives patients of their eating experience and can lead to poor compliance and low appetite (16). A study has suggested that patients' emotional state and willingness to eat are associated with the effectiveness of tube feeding (17). Private caregivers are expected to improve the nutritional status of patients through psychological support and daytime care. However, relevant reports are lacking. By accurately understanding the effect of private caregivers on nutrition and mental health, we can optimize their use, explore ways to provide support in these areas, assess whether they have a positive impact, and add care value. This can help reduce the burden on patients' families and minimize the waste of healthcare resources. Therefore, this observational study aimed to explore the impact of private caregivers on nutritional risk and anxiety in patients with dysphagia after ischemic stroke.

## 2 Methods

This study was approved by the Medical Ethics Committee of The First Affiliated Hospital of Zhengzhou University (2022-KY-0094-002), and all participants signed informed consent forms.

### 2.1 Study design

This observational study was conducted at five hospitals in central China. We invited ischemic stroke survivors with dysphagia who sought treatment in the Department of Rehabilitation and conducted assessments on the first and tenth day of hospitalization. Propensity score matching (PSM) was used to balance confounding factors, including demographic and clinical characteristics (18). The net effect of private caregivers on nutritional risk and anxiety was analyzed in the matched sample.

### 2.2 Participants

The inclusion criteria were: (1) age >18 years. (2) Ischemic stroke (19). (3) Nutritional risk screening-2002 (NRS-2002) ≥3, (20) indicating the need of nutritional supports (4). Based on the patient's worst swallowing performance in modified barium swallow studies, the penetration-aspiration scale (PAS) ≥3 on the videofluoroscopy (21). (5) Duration from onset to admission <15 days. (6) Stable conditions and good consciousness, able to cooperate with treatment and questionnaires. (7) Hospital stays at least 10 days. (8) First stroke.

The exclusion criteria were: (1) other brain diseases, cancers, or severe systemic diseases. (2) Malnutrition or psychiatric disorders before this stroke, based on medical records or evidence from family reports (e.g., pictures or documents). (3) Tracheostomized patients. Participants were retrospectively excluded if they (1) experienced stroke recurrence, (2) used parental nutrition during the study, or (3) had severe deterioration in condition. Participants had the right to quit the study halfway and the dropout cases were excluded from the final analysis.

Before entering the study, the research staff introduced private caregivers in a uniform and unbiased manner. The aims were (1) to minimize the influence of non-research-related factors on the decisions made by patients' families, (2) to ensure the informed consent rights of patients and their families, and (3) ethical considerations. Whether to hire a private caregiver entirely depended on patients and their family and was independent of this study. In this study, all private caregivers were provided by a third party, independent of the hospital and this study. To ensure the homogeneity of private caregiving services, we requested that the private caregivers: (1) were trained by the company for at least 3 months before work, (2) had worked in caregiving for 5–10 years, and (3) did not have a medical background. Patients who hired private caregivers for at least 6 days during the study were included in the caregiver group; otherwise, they were placed in the non-caregiver group. The decision to hire a private caregiver did not affect the duties of doctors and nurses. Due to their lack of medical background, private caregivers did not participate in clinical nursing work.

The scope of the private caregivers' work included the following aspects: (1) morning routine: (a) assisting with personal hygiene: help the patient with brushing teeth, washing face, and other morning hygiene activities. (b) Getting dressed: assist the patient in getting dressed, ensuring they are comfortable and appropriately clothed for the day. (c) Mobility assistance: help the patient get out of bed, move to a wheelchair or chair, and ensure they are positioned safely and comfortably. (2) Meal assistance: (a) monitoring nutrition: keep track of the patient's food and fluid intake, encouraging and help patients to meet their nutritional needs. (3) Medication management: (a) Administering medications: assist the patient in taking prescribed medications at the correct times, ensuring they follow the doctor's instructions. (b) Monitoring side effects: observe the patient for any adverse reactions to medications and report any concerns to the medical staff. (4) Therapy and exercises: (a) rehabilitation exercises: assist the patient with prescribed physical therapy exercises, helping them perform movements correctly and safely. (b) Speech therapy support: encourage and assist the patient with speech therapy exercises, especially if they have difficulty swallowing or speaking. (5) Emotional and psychological support: (a) companionship: spend time talking with the patient, providing emotional support, and engaging in activities that the patient enjoys. (b) Encouragement: motivate the patient to stay positive and adhere to their treatment and rehabilitation plans. (6) Daily living activities: (a) toileting assistance: help the patient with using the toilet, ensuring they maintain dignity and hygiene. (b) Bathing: assist with bathing or showering, ensuring the patient is clean and comfortable. (7) Monitoring health status: (a) observing symptoms: continuously monitor the patient's condition, noting any changes in symptoms or behavior. (b) Reporting to medical staff: communicate any significant observations or concerns to the healthcare team promptly. (8) Evening routine: (a) preparing for bed: assist the patient with their evening routine, including changing into nightwear and settling into bed. (b) Ensuring comfort: make sure the patient is comfortable, adjusting pillows and blankets as needed. (9) Additional responsibilities: (a) transportation: accompany the patient to medical appointments or therapy sessions if needed. (b) Housekeeping: perform light housekeeping tasks to ensure the patient's environment is clean and safe. (c) Emergency preparedness: be prepared to handle emergencies, such as sudden health deteriorations, and know the protocols for contacting medical help. (d) Keeping records: maintain a log of the patient's daily activities, food intake, medication administration, and any notable incidents or changes in condition.

### 2.3 Assessment

#### 2.3.1 Anxiety

The 7-item generalized anxiety disorder (GAD-7) was used to evaluate anxiety on days 1 and 10. This scale has been validated for Chinese inpatients (22). In this study, patients were divided into non-to-mild anxiety (GAD-7 ≤ 9 points) and moderate-to-severe anxiety (GAD-7 > 9 points) groups. For patients with limited comprehension abilities or mild aphasia, researchers were allowed to provide non-leading explanations of the items.

#### 2.3.2 Nutritional risks

The NRS-2002 was used to evaluate nutritional risks on days 1 and 10. It was initially designed for severely ill patients or those with increased nutritional needs or undernutrition, and its adaptability makes it suitable for our study population (23–25). This nutritional risk screening tool is suitable for use over a period of more than 1 week. The assessment included nutritional status and disease severity. The NRS-2002 has been widely validated in stroke survivors in the Chinese context (26). A total score of ≥3 indicated nutritional risk (20).

#### 2.3.3 Covariates

Baseline demographic and clinical characteristics were used as covariates for matching, to ensure comparability. The details and assignments of the covariates are provided in Supplementary Appendix S1. Due to the need for enteral feeding, the feeding type has been included as a covariate. We provided two common types of enteral nutrition support in China: intermittent oro-esophageal tubes (IOE) and nasogastric tubes (NGT). For IOE, the tube was intermittently inserted orally into the upper esophagus (27), whereas for NGT, the tube was placed persistently through the nostril into the stomach (28). The feeding types were selected through negotiation among patients and doctors, independent of this study. According to the relevant guidelines, the nutritional standards were the same, with a daily intake of 25–35 kcal/kg and protein intake of 0.8–1.2 g/kg (29). Independent clinical nutritionists arranged individualized feeding contents for the patients. The patients' actual intake fluctuated around the nutritional standards and was influenced by factors such as compliance, adverse events, and willingness to eat. However, all the staff members made every effort to prevent malnutrition. All feedings were administered by healthcare professionals instead of patients' family members or private caregivers. Secondary prevention with appropriate medication, health education guidance, and rehabilitation interventions were provided individually for all patients.

### 2.4 Statistical analysis

Categorical data, normal continuous data, and skewed continuous data were expressed using counts and percentages (*n*, %), means and standard deviations (*x* ± *s*), and medians and interquartile ranges [*M* (*Q*_25_, *Q*_75_)], respectively. For single-factor analysis, we used chi-square, *t* or rank-sum tests to explore any differences in covariates between the non- and private caregiver groups. The significant items were included in a set of covariates for subsequent PSM. The anxiety on day 1 was included in the PSM to balance baseline situations. In the PSM, the patient groups were set as independent variables, and the GAD-7 and NRS-2002 on day 10 were set as outcomes variables. The average treatment effect on the treated (ATT) values were used to report the associations between independent and outcome variables before and after matching. Propensity scores were used for matching, which were based on logistic regression. We matched the sample 1:1 without replacement (caliper = 0.1). A *P* value <0.05 was considered statistically significant, and SPSS 21.0 was used for statistical analysis.

## 3 Results

### 3.1 Basic information

In total, we invited 2,819 potential participants who met the inclusion criteria. Among them, 332 patients met the exclusion criteria upon admission, 75 patients were retrospectively excluded, and another 178 patients quit the study voluntarily. Finally, 2,234 cases were included in the final analysis. There were 766 patients who hired private caregivers. Before PSM, there were significant differences in anxiety (47.00 vs. 32.86%, *P* < 0.001) and nutritional risks (100.00 vs. 70.55%, *P* < 0.001) on days 1 and 10. Additionally, the male participants showed a significantly higher prevalence of moderate-to-severe anxiety than female participants on day 10 (36.39 vs. 25.04%, *P* < 0.001). There were no significant sex differences in nutritional risk on day 10 (*P* = 0.102). The details are shown in Table 1.

**Table 1:** The anxiety and nutritional risks before matching (*n*, %).

**Time point**	**Group**	**Anxiety**		**Nutritional risks**	

		**None-to-mild**	**Moderate-to-severe**	**Yes**	**No**
Day 1	All participants (*n* = 2,234)	1,050 (47.00)	1,184 (53.00)	2,234 (100.00)	–
Day 10	All participants (*n* = 2,234)	732 (32.86)	1,502 (67.14)	1,576 (70.55)	658 (29.45)
Day 10	Male participants	560 (36.39)	979 (63.61)	1,102 (71.60)	437 (28.40)
Day 10	Female participants	174 (25.04)	521 (74.96)	474 (68.20)	221 (31.80)
Day 10	The private caregiver group (*n* = 766)	175 (22.85)	591 (77.15)	545 (71.02)	221 (28.98)
Day 10	The non-private caregiver group (*n* = 1,468)	559 (38.08)	909 (61.92)	1,032 (70.30)	436 (29.70)

### 3.2 Single-factor analysis

Significant differences were found between the groups in the following items (*P* < 0.05): anxiety on day 1, sex, age, disease course, cigarette use, alcohol intake, hypertension, the PAS, neurological impairment, and feeding modes, as shown in Table 2. These variables were included in the set of covariates for the subsequent PSM.

**Table 2:** Single-factor analysis.

**Items**	**The non-private caregiver group (*n* = 1,468)**	**The private caregiver group (*n* = 766)**	***P*-value**
Anxiety on day 1 [*n* (%)]			<0.001^***^
None-to-mild	909 (61.92)	591 (77.15)	
Moderate-to-severe	559 (38.08)	175 (22.85)	
Sex [*n* (%)]			0.001^**^
Female	492 (33.51)	203 (26.50)	
Male	976 (66.49)	563 (73.50)	
Age [years, *n* (%)]			<0.001^***^
30–40	41 (2.79)	31 (4.05)	
40–50	271 (18.46)	95 (12.40)	
50–60	609 (41.49)	290 (37.86)	
60–70	312 (21.25)	156 (20.37)	
70+	235 (16.01)	194 (25.33)	
Lesion location [*n* (%)]		
Cortex	242 (14.49)	122 (15.92)	0.428
Subcortex	469 (31.95)	254 (33.16)	
Brainstem	664 (45.22)	341 (44.52)	
Cerebellum	93 (6.34)	59 (7.70)	
Disease course [days, *n* (%)]			<0.001^***^
<6	347 (23.64)	86 (11.23)	
6–10	501 (34.13)	209 (27.28)	
>10	620 (42.23)	471 (61.49)	
Marital status [*n* (%)]		
Married	1,292 (88.01)	688 (89.82)	0.201
Others (unmarried, divorced, and widowed)	176 (11.99)	78 (10.18)	
Educational levels [*n* (%)]		
High school and above	516 (35.15)	247 (32.25)	0.169
Blow high school	952 (64.85)	519 (67.75)	
Cigarette use [*n* (%)]			<0.001^***^
Never	615 (41.89)	367 (47.91)	
Quit	349 (23.77)	115 (15.01)	
Yes	504 (34.33)	284 (37.08)	
Alcohol intake [*n* (%)]			<0.001^***^
Never	648 (44.14)	403 (52.61)	
Quit	375 (25.54)	132 (17.23)	
Yes	445 (30.31)	231 (30.16)	
Hypertension [*n* (%)]			0.009^**^
No	421 (28.68)	180 (23.50)	
Yes	1,047 (71.32)	586 (76.50)	
Hyperlipidemia [*n* (%)]		
No	961 (65.46)	505 (65.93)	0.827
Yes	507 (34.54)	261 (34.07)	
Type 2 diabetes [*n* (%)]		
No	1,012 (68.94)	498 (65.01)	0.060
Yes	456 (31.06)	268 (34.99)	
Pneumonia [*n* (%)]		
No	881 (60.01)	475 (62.01)	0.359
Yes	587 (39.99)	291 (37.99)	
Penetration-aspiration scale [levels, *M* (*Q*_25_, *Q*_75_)]	6.00 (5.00, 7.00)	5.00 (5.00, 6.00)	<0.001^***^
Modified Barthel index [points, *M* (*Q*_25_, *Q*_75_)]	55.00 (40.00, 70.00)	55.00 (35.00, 80.00)	0.157
National institutes of health stroke scale [points, *n* (%)]			0.003^**^
0–4	255 (17.37)	109 (14.23)	
5–15	1,052 (71.66)	599 (78.20)	
>15	161 (10.97)	58 (7.57)	
Paralysis [*n* (%)]			0.161
No	342 (23.27)	153 (19.96)	
Hemiplegia	911 (62.05)	488 (63.72)	
Quadriplegia	215 (14.68)	125 (16.32)	
Post stroke visual impairment [*n* (%)]			0.232
No	1,036 (70.58)	559 (73.04)	
Yes	432 (29.42)	207 (26.96)	
Aphasia [*n* (%)]			0.584
No	1,215 (82.76)	641 (83.68)	
Yes	253 (17.24)	125 (16.32)	
Feeding modes [*n* (%)]			0.014^*^
Intermittent oro-esophageal tubes	870 (59.26)	495 (64.62)	
Nasogastric tubes	598 (40.74)	271 (35.38)	

^*^P <0.05, ^**^P <0.01, ^***^P <0.001.

### 3.3 Propensity score matching

**Figure 1:**
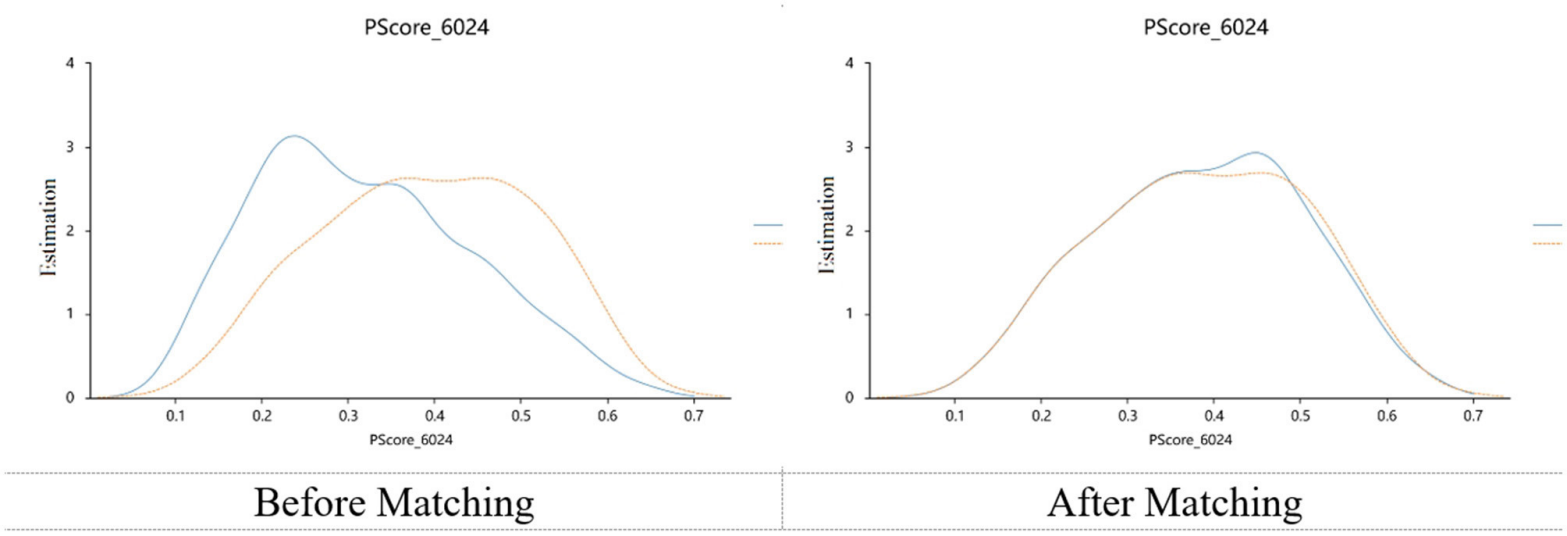
Kernel density plots.

A total of 766 pairs were successfully captured using PSM. There were no significant differences in the matched samples for all covariates, and the absolute values of the standardized bias were all <0.1. This indicated that in the matched sample, the relationship between the independent and dependent variables was not affected by the covariates in the statistical analysis. The PSM balance test is shown in Table 3, and the kernel density plots are shown in Figure 1. The ATT analysis showed that before and after PSM, there was no significant correlation between groups and nutritional risks (30.75 vs. 29.41%, *P* = 0.573), but there was a significant correlation between groups and anxiety on day 10 (34.89 vs. 23.40%, ATT = 0.234 after matching, *P* < 0.001), as shown in Table 4.

**Table 3:** Propensity score matching balance test.

**Item**	**Condition**	**Treated**	**Control**	**Standardized bias (%)**	**Standardized bias reduction (%)**	**Eigen value**	***P*-value**
Anxiety on day 1	Before	0.460	0.475	−3.19	66.42	−0.717	0.473
	After	0.453	0.459	−1.07		−0.208	0.836
Sex	Before	0.735	0.665	15.34	49.99	3.479	0.001
	After	0.729	0.762	−7.67		−1.484	0.138
Age	Before	2.505	2.292	19.80	96.24	4.388	<0.001
	After	2.480	2.488	−0.74		−0.144	0.886
Disease course	Before	2.503	2.186	42.70	99.08	9.792	<0.001
	After	2.484	2.487	−0.39		−0.076	0.940
Cigarette use	Before	1.892	1.924	−3.66	−50.46	−0.816	0.415
	After	1.902	1.952	−5.51		−1.066	0.286
Alcohol intake	Before	1.775	1.862	−9.95	90.64	−2.219	0.027
	After	1.794	1.786	0.93		0.180	0.857
Hypertension	Before	0.765	0.713	11.81	92.08	2.677	0.008
	After	0.759	0.755	0.93		0.181	0.857
The penetration-aspiration scale	Before	5.563	5.867	−25.19	78.95	−5.718	<0.001
	After	5.582	5.643	−5.30		−1.025	0.305
Neurological impairment	Before	0.933	0.936	−0.51	−495.82	−0.118	0.906
	After	0.930	0.916	3.06		0.591	0.555
Feeding modes	Before	0.354	0.407	−11.04	16.77	−2.489	0.013
	After	0.338	0.382	−9.19		−1.778	0.076

**Table 4:** ATT analysis.

**Sample**	**Treated**	**Control**	**ATT value**	**SE**	***t***	***P*-value**
**Anxiety on day 10**						
Unmatched	0.228	0.381	−0.152	0.020	−7.702	<0.001^***^
Matched	0.234	0.349	−0.115	0.023	−4.929	<0.001^***^
**Nutritional risks**						
Unmatched	0.710	0.703	0.007	0.020	0.354	0.724
Matched	0.706	0.693	0.013	0.024	0.563	0.573

^***^P <0.001.

ATT, average treatment effect on the treated.

## 4 Discussion

This observational study aimed to explore the effect of private caregivers on nutritional risk and anxiety among stroke survivors with dysphagia. The primary findings revealed that private caregivers significantly reduced anxiety levels in patients during the 10-day hospitalization but had no significant effect on nutritional risk. This indicated that, while private caregivers can alleviate psychological distress, their influence on nutritional outcomes might be limited, particularly in settings where nutritional support is managed by hospital staff. In addition, this study showed sex differences in the prevalence of post-stroke anxiety. Previous studies have reported that the prevalence of post-stroke psychological disorders is higher in males than in females, which is consistent with our results (30). The study highlighted the potential of private caregivers to complement healthcare resources, particularly in addressing the psychological needs of stroke survivors. However, further research is needed to optimize their role in managing nutritional risks.

The unmatched sample showed that approximately half of the participants experienced anxiety upon admission. After the 10-day rehabilitation intervention, this rate decreased to ~30%, consistent with the findings of previous studies (31). A longitudinal study has indicated that the psychological status of tube-fed patients improved during the rehabilitation period, which is consistent with the current study (32). Dysphagia has been associated with psychological problems (33), and feeding tubes can pose an additional burden on patients (34). The overall nutritional risk ratio of the patients decreased by ~30%, which is consistent with a previous study (35). Dysphagia patients typically receive various interventions in the rehabilitation department, such as nerve block therapy (36), behavioral training (28), and acupuncture (37). Although these therapies are not directly related to nutritional status, they can influence nutritional intake by improving neurological function, mood, and swallowing ability (38, 39). This factors can ultimately contribute to a reduction in nutritional risk.

Population aging has increased the prevalence of stroke and the demand for medical resources (40). The shortage of nursing staff is particularly severe in developing countries. As a compromise, private caregivers in these regions could partially alleviate the burden of care for nurses and family members (41). Their roles are similar to those of the family caregivers (42). However, research on private caregivers is limited. In developed regions, private caregivers typically refer to full-time employment of professional nursing staff (43). This situation has not yet been widely realized in areas such as China and India. Moreover, the scope of work in this profession may vary across different regions (7). In China, the social security system has not yet extended its coverage to private care. In recent years, the challenges posed by an aging society have been gradually acknowledged. Therefore, some organizations and regions have provided subsidies or support for private caregiving, such as caregiver training and public welfare caregiving. However, cost-benefit analyses of caregiving still require further research to assist families and policymakers in understanding the long-term economic benefits of investing in private care (44). This study found that hiring private caregivers can significantly improve anxiety levels in patients with post-stroke dysphagia. Private caregivers are expected to understand the needs and habits of their patients and provide attentive and customized services (45). They can offer round-the-clock care and provide emotional support and companionship to patients, which helps to alleviate anxiety (46). Additionally, private caregivers can assist patients with activities of daily living, reduce their burden, and closely monitor changes in their condition (47). This allows for timely identification of and response to anxiety, thereby providing better care for patients. One study has suggested that family caregiving can increase the psychological burden on patients (48), while having a private caregiver who is not a family member may help reduce anxiety.

Nutrition is fundamental to rehabilitation and dysphagia is closely associated with malnutrition (49). One study indicated that stroke patients with dysphagia had a significantly higher risk of malnutrition than those with normal swallowing function (50). In China, tube feeding is the most common method for enteral nutrition support. The two tube-feeding methods provided in this study are widely applied for dysphagia caused by various diseases (13, 27, 51, 52). We incorporated this variable into the matching process to reduce potential influences caused by the feeding methods, which is one of the innovations of this research. The matched samples were well-balanced across all covariates, and the kernel density plots indicated good matching effectiveness. While some studies have suggested that private caregiving can improve rehabilitation outcomes (13, 41, 53), we did not observe a correlation between private caregivers and malnutrition risk in the matched samples. The focus of private caregivers is typically on providing daily living assistance, rather than specifically managing nutrition (46). Moreover, all tube-feeding operations in this study were carried out by hospital nursing staff, which limited the role of private caregivers. Although we speculated that private caregivers could improve patients' nutritional intake by encouraging them, enhancing compliance, and supporting psychological aspects, this effect remains insignificant. We hypothesized that one of the reasons might be that tube feeding deprived them of their eating experience. The patients could not taste, chew, or actively swallow the food (50). This reduced patients' proactive engagement in nutritional intake. Furthermore, during rehabilitation, patients' nutritional standards were prescribed uniformly. Nursing staff have typically made every effort to help patients achieve the nutritional goals set by dietitians, which may diminish the contributions of private caregivers. It should be noted that in some clinical practices, some private caregivers are authorized to perform tube-feeding procedures. This situation may have a more significant influence on the patients' nutritional status (54). Under this circumstances, nutritional outcomes may be further affected by factors such as the professional experience of caregivers and the relationship between private caregivers and patients.

This study had several limitations. First, we only collected data from patients with dysphagia, which limited the representativeness of the sample. Second, as an observational study, we were unable to standardize the interventions received by different groups. For ethical reasons, we did not restrict the involvement of family members in daily care, which might have introduced potential bias. Third, although we discussed potential mechanisms, we did not collect the mediating variables for statistical analysis. Furthermore, although socioeconomic status has been hypothesized to be associated with private care, income level and social status are often underreported. We did not receive support from the relevant authorities to access such data directly. Consequently, socioeconomic status was not included as a covariate in the PSM. Additionally, due to the confidentiality of patient data and the difficulty in achieving cross-regional collaboration, we have only collected data from several hospitals in central China. This affected the representativeness of the results and feasibility of cross-cultural comparisons. Also, we did not explore long-term effects. The primary reason was that the average length of hospital stays for patients in the study hospital did not exceed 14 days. As the observation period increased, the sample size decreased significantly. Moreover, patients no longer needed private caregivers after discharge. This raised concerns that uncontrolled variables in their post-discharge lives might introduce greater bias. A large-scale long-term cohort study should be designed to address these issues. Finally, although we used PSM to reduce confounding factors. The observational design cannot fully establish causality unless a randomized controlled study is conducted. Therefore, this study only revealed the predictive relationships between the variables. Stronger evidence is necessary to establish causality.

## Data Availability

The raw data supporting the conclusions of this article will be made available by the authors, without undue reservation.
