# Supplementary material for: Effects of private caregivers on nutritional risk and anxiety in stroke survivors with dysphagia: an observational study
Source: Front Nutr. 2025 May 30;12:1513609. doi: 10.3389/fnut.2025.1513609 (PMC12162323; doi:10.3389/fnut.2025.1513609)
Supplement: Supplementary file 1 [file Table_1.docx]

Appendix 1. The List of Covariates

| Variables | Definition | Assignment |
| --- | --- | --- |
| Sex | Based on the Chinese resident identity card, rather than self-identified gender. | Male=1; Female=0 |
| Age | Calculated based on the date of birth from the Chinese resident identity card. | =age |
| Lesion location | Based on the imaging data from this stroke. | dummy coding yes=1, no=0 for “Cortex; Subcortex; Brainstem; Cerebellum” |
| Disease course | The period from the onset to admission to the rehabilitation department. | dummy coding yes=1, no=0 for “<6, 6-10, >10” |
| Marital status | Based on self-reports or family reports. | Married=1, Others (unmarried, divorced and widowed)=0 |
| Educational level | Based on self-reports or family reports. | High school and above=0. Blow high school=1 |
| Cigarette use | Based on self-reports or family reports, smoking more than once a week continuously for six months is considered yes; having smoked but not smoking for more than six months is considered quitting. | dummy coding yes=1, no=0 for “Never, Quit, Yes” |
| Alcohol intake | Based on self-reports or family reports, drinking more than once a week continuously for six months is considered yes; having drunk but not drinking for more than six months is considered quitting. | dummy coding yes=1, no=0 for “Never, Quit, Yes” |
| Hypertension | Based on self-reports, family reports, or medical records. | Yes=1; No=0 |
| Hyperlipidemia | Based on self-reports, family reports, or medical records. | Yes=1; No=0 |
| Type 2 diabetes | Based on self-reports, family reports, or medical records. | Yes=1; No=0 |
| Pneumonia | Based on the imaging data from this stroke or the doctor's diagnosis. | Yes=1; No=0 |
| National Institutes of Health Stroke Scale | Assessment by healthcare professionals. | =scores |
| Paralysis | Significant differences in strength between sides or weakness in all four limbs. | dummy coding yes=1, no=0 for “No, Hemiplegia, Quadriplegia” |
| Post-stroke visual impairment | Including hemianopia, visual neglect, diplopia, double vision, etc. | Yes=1; No=0 |
| Modified Barthel Index | Assessment by healthcare professionals. | =scores |
| Aphasia | Assessment by healthcare professionals. | Yes=1; No=0 |
| Penetration-Aspiration Scale | In the modified barium swallow study, the patient swallows different textures of contrast food boluses from less to more, and is assessed under Videofluoroscopic Swallowing Study. | =scores |
| Feeding | IOE or NGT | IOE=1; NGT=0 |
